# Improved Antitumor Efficacy of Combined Vaccine Based on the Induced HUVECs and DC-CT26 Against Colorectal Carcinoma

**DOI:** 10.3390/cells8050494

**Published:** 2019-05-22

**Authors:** Qiushuang Zhang, Chao Xie, Dongyu Wang, Yi Yang, Hangfan Liu, Kangdong Liu, Jimin Zhao, Xinhuan Chen, Xiaoyan Zhang, Wanjing Yang, Xiang Li, Fang Tian, Ziming Dong, Jing Lu

**Affiliations:** 1 Department of Pathophysiology, School of Basic Medicine, Zhengzhou University, Zhengzhou 450001, Henan, China; zhangqiushuang58@163.com (Q.Z.); xiechao_9966@126.com (C.X.); wdy1028796652@163.com (D.W.); yangyi20086@163.com (Y.Y.); m15249680100@163.com (H.L.); kdliu@zzu.edu.cn (K.L.); zjm.0427@163.com (J.Z.); chen_xinhuan@126.com (X.C.); zhangxiaoyan@zzu.edu.cn (X.Z.); ywj1102@zzu.edu.cn (W.Y.); ninika5009@126.com (X.L.); tianfang418@163.com (F.T.); dongzm@zzu.edu.cn (Z.D.); 2 Collaborative Innovation Center of Henan Province for Cancer Chemoprevention, Zhengzhou 450001, Henan, China

**Keywords:** angiogenesis, human umbilical vein endothelial cell, vaccine, tumor microenvironment, dendritic cell, colorectal carcinoma

## Abstract

Angiogenesis is essential for the development, growth, and metastasis of solid tumors. Vaccination with viable human umbilical vein endothelial cells (HUVECs) has been used for antitumor angiogenesis. However, the limited immune response induced by HUVECs hinders their clinical application. In the present study, we found that HUVECs induced by a tumor microenvironment using the supernatant of murine CT26 colorectal cancer cells exerted a better antiangiogenic effect than HUVECs themselves. The inhibitory effect on tumor growth in the induced HUVEC group was significantly better than that of the HUVEC group, and the induced HUVEC group showed a strong inhibition in CD31-positive microvessel density in the tumor tissues. Moreover, the level of anti-induced HUVEC membrane protein antibody in mouse serum was profoundly higher in the induced HUVEC group than in the HUVEC group. Based on this, the antitumor effect of a vaccine with a combination of induced HUVECs and dendritic cell-loading CT26 antigen (DC-CT26) was evaluated. Notably, the microvessel density of tumor specimens was significantly lower in the combined vaccine group than in the control groups. Furthermore, the spleen index, the killing effect of cytotoxic T lymphocytes (CTLs), and the concentration of interferon-γ in the serum were enhanced in the combined vaccine group. Based on these results, the combined vaccine targeting both tumor angiogenesis and tumor cells may be an attractive and effective cancer immunotherapy strategy.

## 1. Introduction

Colorectal carcinoma (CRC) is one of the most common and deadly cancers worldwide, and its incidence is on the rise. About 1.09 million new cases were diagnosed, and more than 550,000 people died from the disease in 2018 [1]. Tumor angiogenesis is required for cancer growth and metastasis and has been considered a potential target for CRC treatment [2]. At present, most antiangiogenic drugs, such as synthetic molecules and monoclonal antibodies, have obvious side effects and readily produce drug resistance, which limits their clinical application [3]. Currently, immunotherapy of tumors with vaccines is another promising avenue of antiangiogenesis therapy to overcome these drawbacks [4,5,6].

Recent studies have demonstrated that a human umbilical vein endothelial cell (HUVEC) vaccine could inhibit tumors by initiating antiangiogenic effects with both cellular and humoral immunity [7,8,9,10]. Presenting various growth factors during angiogenesis, endothelial cell vaccines may be more effective than targeting one specific antigen in tumor blood vessels. Antitumor effects of endothelial cell vaccines have been demonstrated in preclinical mouse models and clinical trials [7,9,10,11,12,13]. Cultured HUVECs in vitro are proliferative endothelial cells, similar to new vessels with proliferative activity in solid tumors and express some proteins absent or barely detectable in quiescent vascular endothelium [14]. However, the immune response induced by a HUVEC vaccine is limited because HUVECs are still very different from tumor endothelial cells. One of the biggest challenges of using a HUVEC vaccine is how to improve the effectiveness of antitumor therapy. Here, we hypothesized that HUVECs induced by the tumor microenvironment might have characteristics more like tumor vascular endothelial cells than HUVECs, thereby producing a stronger suppressive effect on tumor angiogenesis. To test this hypothesis, we obtained the supernatant of murine CT26 colorectal carcinoma cells to simulate a tumor microenvironment and investigated its influence on HUVECs.

Dendritic cells (DCs) are the most powerful specialized antigen-presenting cells in vivo. These cells can take up tumor antigen and induce cytotoxic T lymphocytes (CTLs) to mediate powerful specific antitumor immune effects [15]. During the last few decades, researchers have achieved inhibitory tumor effects with DCs loaded with tumor-associated antigens, cytokines, chemokines, and other modifications by activating the antitumor immune response [16,17,18,19,20]. By incubating DCs with whole tumor lysates or autologous tumor cells, a higher number of antigens can be obtained, which can express multiple epitopes on MHC class I or II, leading to T and cytotoxic reactions. DCs-based vaccines have shown promising results in terms of safety and immunogenicity in both preclinical and clinical settings [21,22]. DCs-based vaccines show immunogenicity in the context of human papilloma virus cervical [23], ovarian cancer [24], and colorectal cancer [25,26], so they have attracted much attention. However, the antitumor efficacy of a single DC vaccine is limited.

In this study, first, we demonstrated that an induced HUVEC vaccine had a more powerful antiangiogenesis effect than a HUVEC vaccine. Based on it, an induced HUVEC vaccine was combined with a DC vaccine loading CT26 antigen. The combined vaccine examined here, targeting both tumor vascular endothelial cells and tumor cells, could be used as a new vaccine strategy for cancer therapy.

## 2. Materials and Methods

### 2.1. Animals and Cell Lines

Female BALB/c mice (4–6 weeks old) of SPF grade were purchased from Beijing Vital River Experimental Animal Center (Beijing, China) and reared in a barrier system. Primary HUVECs were obtained from the aseptic cords, which were contributed by healthy parturient donors from the Third Affiliated Hospital of Zhengzhou University. The culture methods were reported previously [27]. This study was approved by the Ethical Committee of Zhengzhou University, and all the experiments performed on mice were conducted in accordance with the guidelines set by the Animal Ethics Committee of Zhengzhou University. The murine CT26 colorectal carcinoma cell line was maintained in our laboratory and cultured in DMEM (Biological Industries, Israel) supplemented with 10% FBS. HUVECs were cultured in an endothelial cell medium (ECM) with 5% FBS (ScienCell, Carlsbad, CA, USA). All of the cell lines were maintained at 37 °C with 5% CO2, and mycoplasma contamination was regularly analyzed in the laboratory.

### 2.2. DCs Generation from Mouse Bone Marrow

The primary bone marrow DCs were extracted from mice bone marrow precursors based on previously reported methods [28,29]. In brief, the tibias and femurs from 6 to 8 week-old female BALB/c mice were flushed to gain bone marrow and then erythrocytes were depleted using commercial lysis buffer (Solarbio, Beijing, China). Cells were washed twice using serum-free RPMI-1640 medium (Biological Industries, Kibbutz Beit Haemek, Israel) and cultured with RPMI-1640 medium which was supplemented with 10% (*v*/*v*) FBS, 10 ng·mL^−1^ recombinant murine GM-CSF (R&D Systems, Minneapolis, MN, USA) and 10 ng·mL^−1^ recombinant murine IL-4 (Peprotech, Rocky Hill, NJ, USA) in six-well plates (1 × 10^6^ cells·mL^−1^; 2 mL·well^−1^) at 37 °C with 5% CO2. Half of the medium was updated with fresh cytokines containing rmGM-CSF and rmIL-4 without discarding any cells on days 3 and 5. On day 7, LPS (Solarbio, Beijing, China) (1 μg·mL^−1^) was added to the medium. Then, DCs were prepared and then identified for use on day 8.

### 2.3. Preparation of the CT26 Cell Culture Supernatant

After reaching 70–80% confluence, the CT26 cells were filled with 5 mL fresh RPMI-1640 medium containing 10% FBS. After 24 h of incubation, the supernatant was collected and centrifuged and then stored at −20 °C.

### 2.4. Preparation of the HUVEC Vaccine

Fixed HUVEC vaccine was prepared with 0.025% glutaraldehyde (*v*/*v*). The concentration of it in PBS was adjusted to 2.5 × 10^7^ cells·mL^−1^ and then stored at −80 °C for injection.

### 2.5. Preparation of the Induced HUVEC Vaccine

The tumor conditioned medium (TCM) was comprised of 60% CT26 cell supernatant and 40% ECM (with 2% FBS in the medium, without growth factors and double antibiotics). For the induced HUVEC group, after reaching 60% confluence, HUVECs were induced by the foregoing TCM for 48 h. A fixed induced HUVEC vaccine was prepared with 0.025% glutaraldehyde (*v*/*v*). The concentration of it in PBS was adjusted to 2.5 × 10^7^ cells·mL^−1^ and then it was stored at −80 °C for injection.

### 2.6. Preparation of the DC-CT26 Vaccine

The CT26 cells were washed twice in PBS carefully and detached with a cell scraper, and then collected in EP tubes. After centrifuging at 530× *g* for 5 min at 4 °C and discarding the supernatant, the cells were resuspended in PBS to adjust the concentration to 1 × 10^7^ cells·mL^−1^. The cells were then encapsulated in cryopreservation tubes. The cell suspensions were centrifuged at 97× *g* for 10 min at 4 °C and filtered through a 0.22 μm filter after they were frozen in liquid nitrogen and disrupted by four freeze-thaw cycles. The supernatant was used as a CT26 freeze-thaw whole antigen. The CT26 cell lysate was removed from the −80 °C freezer and placed at 37 °C for thawing. On the 5th day of DC culture, the CT26 cell lysate (100 μg·mL^−1^) was added to the culture medium. Then, the DC-CT26 vaccine was collected and prepared for immunization.

### 2.7. Vaccination Protocols in Tumor Models

Thirty or forty female BALB/c mice (4–6 weeks old) of SPF grade were randomly divided into three or four groups. In the armpit lymph node area, all mice were immunized with the corresponding vaccine weekly for five consecutive weeks. No blinding was done for the animal studies. Mice were injected with 1 × 10^5^ CT26 tumor cells subcutaneously in their left flank after the last immunization 1 week. When the subcutaneous tumors became palpable, tumor growth was measured every other day. Using the formula V = 0.5ab^2^, the volume was computed with “a” as the long diameter in millimeters and “b” as the short diameter in millimeters. The spleen tissues of mice in each group were peeled, then weighed and photographed. To examine immune function of the body, the spleen index was calculated.

The Spleen Index = The Spleen Weight/Average Weight of Mice(1)

The tumor inhibition rate was computed according to the following formula:

Tumor Inhibition Rate = (Average Tumor Weight in the Control Group − Average Tumor Weight in the Experimental Group) / Average Tumor Weight in the Control Group × 100%(2)

### 2.8. Wound-Healing Assay

HUVECs were cultured in different concentrations (0%, 40%, and 60%) of TCM at 37 °C and 5% CO2 for 48 h. Then, they were seeded in 12-well plates and cultured overnight. After reaching about 90% confluence, the cell monolayer was scratched carefully with a 200 μL pipette tip and then a straight wound was drawn in each well. Each well was washed twice with PBS. At specific time points (0, 24, and 48 h), the injured areas were captured with a microscope (Olympus, Tokyo, Japan). The number of migrated cells per field of each group was counted.

### 2.9. Transwell Assay

Precoating with diluted Matrigel (1:4; BD Biosciences, San Jose, CA, USA) in the membrane surfaces, transwell chambers (pore size: 8 μm; Corning, NY, USA) were incubated for 2 h at 37 °C. HUVECs without serum were plated onto the upper chamber, and the lower chamber was full of a complete endothelial cell medium. After that, the chambers were cultured for 24 h at 37 °C. The invaded cells were fixed with 10% TCA for 1 h and stained with crystal violet for 0.5 h. At last, cells were captured using an inverted microscope (×200) (Olympus, Tokyo, Japan) and counted.

### 2.10. Immunohistochemistry and H&E Staining

Tumor microvessel density was detected by immunostaining with CD31 (ab28364; 1:200; Abcam, Cambridge, UK.). The tumor tissues of each group were fixed with 10% formalin immediately after exsection. Paraffin embedding was performed, and then the tissues were cut into 4 μm slices. Primary antibody was added to incubate with the paraffin slices of tumor tissues at 4 °C overnight. Under high-power fields (×100), the mean microvessel density (MVD) for six fields was counted to show the number of vessels. In correspondence with standard histological procedures, the tumor sections were stained with hematoxylin-eosin (H&E). The results were imaged using the optical microscope.

### 2.11. Detection of the Anti-Induced HUVEC Membrane Protein Antibody by ELISA

With a Membrane and Cytosol Protein Extraction Kit (Beyotime, Shanghai, China), induced HUVEC membrane protein was derived following the manufacturer’s instructions. The protein was diluted in 0.05 mM carbonate-bicarbonate buffer (pH = 9.6), then applied to ELISA plates (JetBioFil, Guangzhou, China) at 100 μL per well (70 μg·mL^−1^) overnight at 4 °C. After being washed and blocked, the plates were incubated with mice serum samples of each group at a 1:20 dilution at 37 °C for 1 h. Once the incubation with HRP-conjugated rabbit anti-mouse IgG at 37 °C for 30 min was completed, the reaction was finished using TMB (Solarbio, Beijing, China) and stopped with H2SO4 (2 mol·L^−1^). Using an ELISA plate reader (Thermo Scientific, Waltham, MA, USA), the OD 450 nm values were detected. Each sample in the assay was implemented in triplicate.

### 2.12. Hemoglobin Assay

All the blood vessel formation of tumors was measured. In brief, Drabkin’s reagent (Sigma-Aldrich, Inc., St. Louis, Missouri, USA) was applied to examine the content of hemoglobin in the invaded vessels in line with the manufacturer’s instructions. After weighing and homogenizing the tumor tissue in 1 mL Drabkin reagent and centrifuging for 20 min at 12,000× *g*, the supernatant was filtered through a 0.22 μm Millipore filter. Using an ELISA plate reader (Thermo Scientific), the absorbance at 540 nm was detected to test the hemoglobin concentration of samples. The results of the experimental groups were compared with the control group.

### 2.13. Western Blot

Protein extracts were prepared using lysis buffer for Western blot. A BCA protein assay kit (Beyotime, Shanghai, China) was implemented to detect the protein concentration. Equal amounts of the supernatant protein (50 μg) were separately subjected to 10% SDS-PAGE and transferred onto a PVDF membrane (Bio-Rad, Hercules, CA, USA). Primary antibodies were incubated overnight at 4 °C with polyclonal antibodies against VEGFR2, TEM1, TEM8, and β-actin. Antibodies against TEM1 (sc-377221; 1:250) and β-actin (sc-8432; 1:1000) were gained from Santa Cruz Biotechnology (Santa Cruz, Dallas, TX, USA). Antibodies against TEM8 (ab21269; 1:250) and VEGFR2 (ab5473; 1:250) were purchased from Abcam (Cambridge, UK). After hybridization with a horseradish peroxidase-conjugated secondary antibody, blots were visualized using a chemiluminescence detection kit (Beyotime, Shanghai, China).

### 2.14. Flow Cytometry

To detect the CD3^+^CD8^+^ T lymphocytes produced by the spleen and infiltrated in the tumor tissues, T lymphocytes were harvested from immunized mice. After the lysis of erythrocyte and passage through a 70 μm filter, the purified splenic T cells (5 × 10^6^ cells·mL^−1^) were labeled with FITC-labeled anti-CD3e (Clone 145-2C11, Biolegend, San Diego, CA, USA) and PE-labeled anti-CD8a (Clone 53-6.7; Biolegend, San Diego, CA, USA) for 60 min at room temperature. After washing twice with PBS and resuspending in 1 mL PBS with 10% FBS, the stained cells were analyzed by flow cytometry (FCM). The number of CD3^+^CD8^+^ T cells was then quantified using a FACSCalibur with CellQuest software version 5.1 (BD Biosciences, Franklin Lakes, NJ, USA).

### 2.15. Measurement of Cytokines

To detect the concentration of IFN-γ, blood was collected in EP tubes and placed for 2 h at room temperature, then stored for 15 h at 4 °C. The serum samples were centrifuged at 1000× *g* at 4 °C for 5 min. The concentration of IFN-γ in the supernatant was detected using commercially available ELISA kits (ExCell Biotech (Taicang) Co., Ltd, China) in correspondence with the manufacturer’s directions.

### 2.16. Cytotoxic T-Lymphocyte (CTL) Killing Assay

Following the manufacturer’s instructions, CTL assay against CT26 cells was implemented with a CytoTox 96 Non-Radioactive Cytotoxicity Assay kit (Promega, Madison, WI, USA). Briefly, spleen T lymphocytes were isolated from mice of each group by Mouse Spleen Lymphocyte Separation Kit (Solarbio, Beijing, China) after being sacrificed. The T lymphocytes were applied as effectors to be incubated with CT26 cells in a 96-well plate at a 50:1 ratio of effectors for 4 h, and then the absorbance values were detected at 492 nm. At last, the percentage of lysis efficiency was calculated in line with the following formula:

The Percentage of Lysis Efficiency = (Experimental Release − Effectors Spontaneous Release − Target Spontaneous Release)/(Target Maximal Release − Target Spontaneous Release) × 100 %(3)

### 2.17. Statistical Data Analysis

Data were shown as the mean ± SD. In this study, one-way ANOVA and least standard difference (LSD) post hoc test were applied to perform statistical analyses and significance using SPSS v17.0 (SPSS Inc., Chicago, IL, USA), when data were normally distributed. For animal studies, sample size of six mice in each group was estimated and the mice were grouped randomly to ensure power with statistical confidence. In all comparisons *p* < 0.05 was deemed to be statistically significant (*p < 0.05, ** *p* < 0.01, *** *p* < 0.001).

## 3. Results

### 3.1. HUVECs Induced by 60% CT26 Cell Supernatant Had Characteristics Similar to Tumor Vascular Endothelial Cells

**Figure 1:**
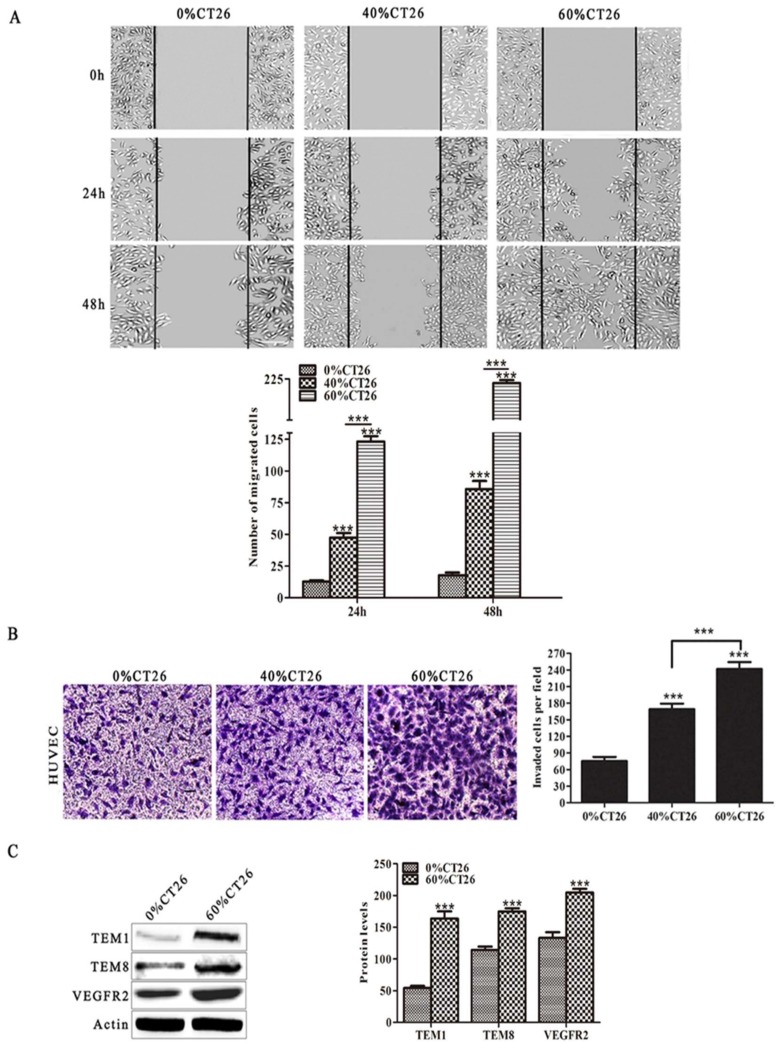
HUVECs induced by 60% CT26 cell supernatant enhanced the capacity of migration and invasion and highly expressed tumor vascular endothelial cell markers. (**A**) The wound-healing test was performed after HUVECs were induced for 48h (scale bar 50 μm); (**B**) the transwell assay was done to examine the invasion ability of HUVECs induced by 0, 40, and 60% CT26 cell supernatant. The number of invaded cells was counted in three random fields. Representative images of invaded cells are shown (scale bar 50 μm); (**C**) Western blot was used to show the expression of the tumor endothelial cell markers TEM1, TEM8 and VEGFR2. Data from three independent experiments were expressed as the mean ± SD, (* *p* < 0.05; ** *p* < 0.01; *** *p* < 0.001).

First, to simulate the tumor microenvironment, different concentrations of TCM (0%, 40% and 60% CT26 cell supernatant) were applied in this study. As migration and invasion are essential for the formation of new blood vessels, wound healing and transwell assays were performed to examine the effects of the tumor microenvironment on the migration and invasion abilities of HUVECs. Notably, the results revealed that the 60% CT26 cell supernatant group had the highest number of migratory and invasive endothelial cells compared with the 0% and 40% CT26 cell supernatant groups (*p* < 0.001 for both) (Figure 1A,B). Furthermore, the expression levels of tumor endothelial cell markers TEM1, TEM8, and VEGFR2 were investigated. Results revealed that the expression of all these markers was markedly higher in HUVECs after induction with the 60% CT26 cell supernatant than in the 0% CT26 cell supernatant group (*p* < 0.001) (Figure 1C).

Next, to exclude the influence of hormones in the serum of the colorectal carcinoma conditioned medium, certified charcoal-stripped FBS (hormone-depleted) was used to collect the colorectal carcinoma cell supernatant. The results showed that conditioned medium of human and murine colorectal cancer cells (hormone-depleted) also promoted the abilities of migration and invasion of HUVECs (*p* < 0.001 for all) (Supplementary Figures S1 and S2).

These results demonstrate that induced HUVECs have characteristics more like tumor vascular endothelial cells than HUVECs. Therefore, we hypothesized that the antitumor effect of an induced HUVEC vaccine might be better than that of a HUVEC vaccine.

### 3.2. HUVEC Vaccine Induced by TCM Elicited a Better Antitumor Effect Than the HUVEC Vaccine

**Figure 2:**
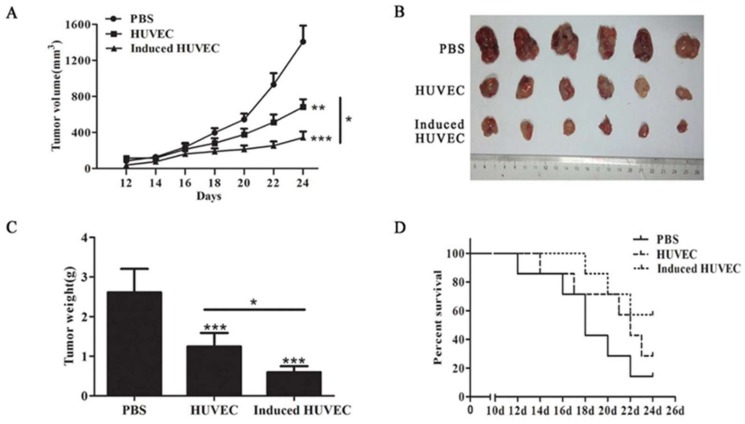
The TCM-induced HUVEC vaccine possessed better antitumor effects than the HUVEC vaccine. (**A**) Tumor growth was measured every other day after day 12; (**B**) tumors of each group were photographed after being stripped from mice; (**C**) tumor weight was tested after removal from mice. (**D**) Survival data of each group was compared, (* *p* < 0.05; ** *p* < 0.01; *** *p* < 0.001).

Fixed xenogeneic endothelial cells have been applied as a vaccine in the immunotherapy of tumors such as lung cancer, hepatoma, and colorectal carcinoma [8,12,30]. To inspect the antitumor effect of HUVECs induced by TCM, we adopted the murine colorectal carcinoma animal model. After immunization, 1 × 10^5^ CT26 cells were injected subcutaneously into mice. Tumor dimensions were measured every other day after day 12. The tumor volume in the induced HUVEC group was significantly lower than that in the HUVEC group (*p* < 0.05) (Figure 2A). Mice were executed on day 24, and the tumors were stripped (Figure 2B). The tumor weight in the induced HUVEC group was significantly lower than that in the HUVEC group (*p* < 0.05) (Figure 2C). Meanwhile, survival monitoring experiment showed that induced HUVEC vaccine prolonged survival of tumor-bearing mice to some extent (Figure 2D). These results suggest that HUVEC induced by TCM has a better suppressive effect on tumor growth than the HUVEC vaccine.

### 3.3. HUVEC Vaccine Induced by TCM Inhibited Tumor Angiogenesis

**Figure 3:**
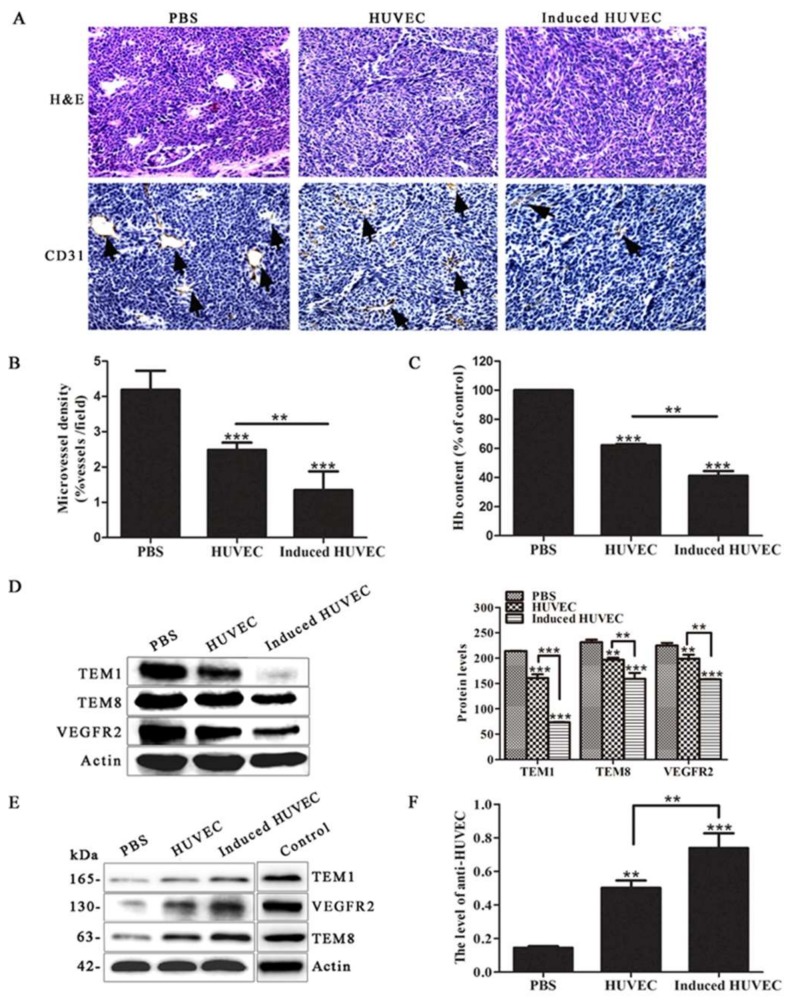
Induced HUVECs exerted better antiangiogenesis effects than the HUVEC vaccine. (**A**) H&E staining and CD31-positive microvessels were tested by immunohistochemical staining (An arrow represented a CD31-positive microvessel); (**B**) vascular density was counted with CD31; (**C**) the hemoglobin level of the tumor tissues was examined by hemoglobin assay; (**D**) Western blot assay was performed to show the levels of the tumor endothelial cell markers TEM1, TEM8, and VEGFR2 in the tumor tissues; (**E**) a Western blot assay was performed to show the levels of anti-TEM1, anti-TEM8, and anti-VEGFR2 in mouse serum; (**F**) the level of the anti-induced HUVEC membrane protein antibody was checked by ELISA, (* *p* < 0.05; ** *p* < 0.01; *** *p* < 0.001).

To verify the mechanism of the better suppressive effect of the induced HUVEC vaccine, the angiogenesis status of tumor samples was investigated by immunohistochemistry. The results showed that the induced HUVEC group exhibited a significantly lower CD31-positive microvessel density in paraffin sections of tumor tissues than the PBS and HUVEC groups (*p* < 0.001, *p* < 0.01, respectively) (Figure 3A,B). Meanwhile, the level of angiogenesis was evaluated from the hemoglobin content in the tumor tissues. The results revealed that HUVECs induced by TCM inhibited tumor angiogenesis markedly in vivo compared with PBS and HUVECs (*p* < 0.001, *p* < 0.01) (Figure 3C). By detecting the expression of tumor endothelial cell markers TEM1, TEM8, and VEGFR2, the results demonstrated that protein levels from the tumor tissues in the induced HUVEC group were significantly decreased compared with the levels from the tissues in the PBS and HUVEC groups (Figure 3D).

In addition, the mouse serum of each group was extracted and applied as a primary antibody for incubation with the total protein of induced HUVECs. Because the mouse serum samples were too limited to incubate with the whole membrane, we had to cut the membrane to only check the expression of objective bands we concerned. Positive bands at the levels of 165, 130, and 63 kDa, like the bands produced by the incubation of TEM1, VEGFR2, and TEM8 antibodies with the total protein of induced HUVECs, were exhibited in the results. The positive bands were therefore presumed to be TEM1, VEGFR2, and TEM8 (Figure 3E). The testing method of anti-HUVEC antibody level induced by HUVEC vaccine and related vaccines has been reported previously [8,12,31]. In this study, the level of anti-induced HUVEC membrane protein antibody was examined by an ELISA assay. Because most of the tumor endothelial cell markers were expressed in the surface, such as TEM1, TEM8, and VEGFR2, we extracted induced HUVEC membrane protein to be coated in 96-well plates [32]. The level of the anti-induced HUVEC membrane protein antibody was examined in mouse serum. The results showed a significantly higher level in the induced HUVEC group than in the PBS and HUVEC groups (Figure 3F), which indicating that induced HUVEC vaccine can elicit a stronger antitumor effect by humoral immunity. These results reveal that immunizing mice with induced HUVECs exerts a better anti-angiogenesis effect than the HUVEC vaccine.

### 3.4. Combined Vaccine of DC-CT26 with Induced HUVECs Enhanced the Antitumor Effect

**Figure 4:**
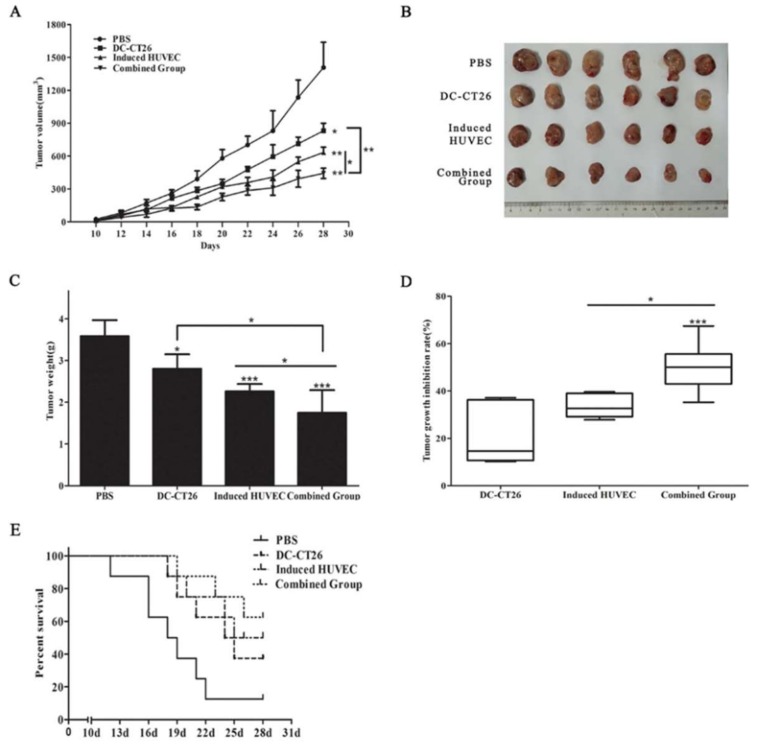
The combination of DC-CT26 with induced HUVECs possessed better antitumor effects than the two single-vaccine groups. (**A**) Tumor growth was measured every other day after day 10. (**B**) Tumors of each group were photographed after being stripped from mice. (**C**) Tumor weight was tested after removal from mice. (**D**) Tumor growth inhibition rate was calculated from the results of tumor weight. (**E**) Survival data of each group was compared, (* *p* < 0.05; ** *p* < 0.01; *** *p* < 0.001).

Previous studies have shown that DC vaccines can activate T lymphocytes to kill tumor cells. Therefore, we expected a better antitumor effect of an induced HUVEC vaccine combined with a DC-CT26 vaccine by targeting tumor vascular endothelial cells as well as tumor cells. To test this assumption, mice were immunized with the corresponding vaccines. Results revealed that the speed of tumor growth in the combined group was slower than that in the two single-vaccine groups (*p* < 0.01, *p* < 0.05) (Figure 4A). Mice were executed on day 28, and the tumors were stripped (Figure 4B). The tumor weight showed a similar pattern (*p* < 0.05 for both) (Figure 4C). Simultaneously, the inhibition rate of tumor growth in the combined group was significantly greater than that in the DC-CT26 group and the induced HUVEC group (*p* < 0.001, *p* < 0.05) (Figure 4D). Mice in the combined vaccine group survived longer (Figure 4E). These results imply that the combination of DC-CT26 with induced HUVECs produces a better antitumor effect than a single-component vaccine alone.

### 3.5. Combined Vaccine of DC-CT26 and Induced HUVECs Exerted a Better Antitumor Angiogenesis Effect Than DC-CT26 or Induced HUVECs Alone

**Figure 5:**
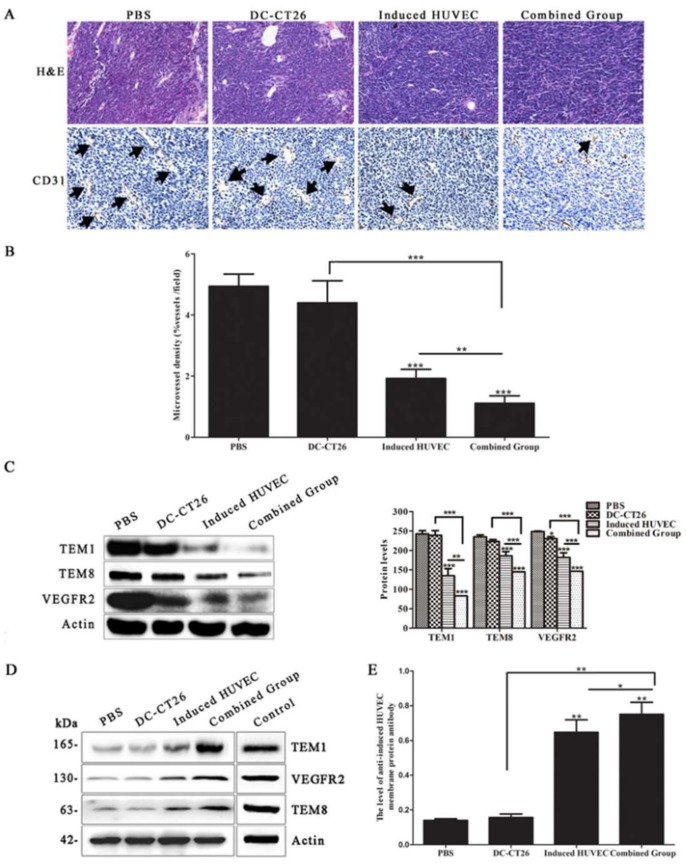
The combined vaccine of DC-CT26 with induced HUVECs exerted better antitumor angiogenesis effects. (**A**) H&E staining and CD31-positive microvessels were tested by immunohistochemical staining; (an arrow represents a CD31-positive microvessel); (**B**) vascular density was counted with CD31-positive staining regions; (**C**) Western blot assay was performed to show the level of the tumor endothelial cell markers TEM1, TEM8, and VEGFR2 in the tumor tissues; (**D**) Western blot assay was performed to show the levels of anti-TEM1, anti-TEM8, and anti-VEGFR2 in mouse serum; (**E**) the level of anti-induced HUVEC membrane protein antibody was checked by ELISA, (* *p* < 0.05; ** *p* < 0.01; *** *p* < 0.001).

To explore the mechanism of the better effect of the combined group, the status of angiogenesis was evaluated by immunohistochemistry. The results revealed that the microvessel density of the combined group was obviously less than that of the DC-CT26 group and the induced HUVEC group (*p* < 0.001, *p* < 0.01, respectively) (Figure 5A,B). Because DCs were reported to directly interact with B lymphocyte to induce germinal center and antibody responses, the anti-angiogenesis effect of the combined vaccine might be promoted by the enhanced humoral immunity response [33]. By analyzing expression of the tumor vascular endothelial cell markers TEM1, TEM8, and VEGFR2 in the tumor tissues, the results demonstrated that angiogenesis in the experimental groups, especially in the combined group, was significantly lower than that in the two single-vaccine groups (Figure 5C).

Furthermore, the mouse serum of each group was applied as a primary antibody to incubate with the total protein of induced HUVECs. Positive bands were detected at the levels of 165, 130, and 63 kDa by western blot, like bands produced by the incubation of TEM1, VEGFR2, and TEM8 antibodies with the total protein of induced HUVECs. The positive bands were therefore presumed to be TEM1, VEGFR2, and TEM8 (Figure 5D). Meanwhile, the level of anti-induced HUVEC membrane protein antibody in mouse serum was determined by an ELISA assay. The results revealed that the level of anti-induced HUVEC membrane protein antibody in the serum was obviously higher in the combined group than in the DC-CT26 group and the induced HUVEC group (*p* < 0.01, *p* < 0.05, respectively) (Figure 5E), indicating that the DC-CT26 vaccine can enhance the humoral immunity induced by the induced HUVEC vaccine. These results suggest that immunizing mice with the combined vaccine of DC-CT26 and induced HUVECs produces a better antitumor effect than the two single-vaccine groups by suppressing angiogenesis.

### 3.6. Combined Vaccine of DC-CT26 with Induced HUVECs Increased the Immune Function of the Spleen

**Figure 6:**
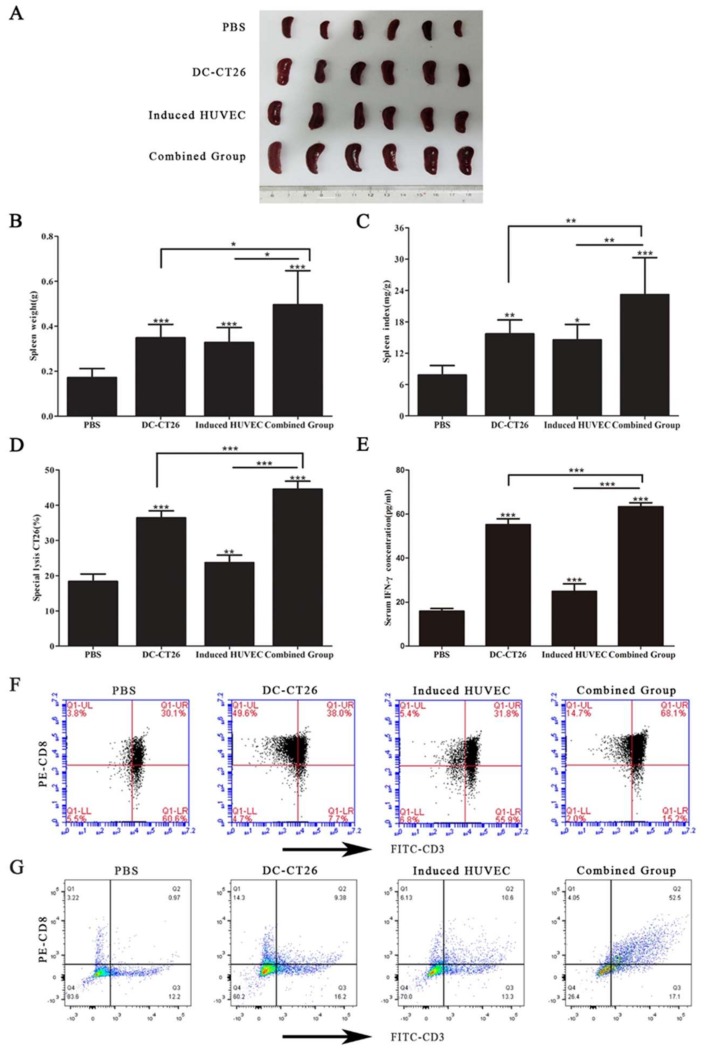
Immunization with DC-CT26 and induced HUVECs increased the immune function of the spleens. (**A**) The picture of the spleens was taken after removal from the mice; (**B**) the weight of the spleens was recorded; (**C**) the spleen index was calculated; (**D**,**E**) the lysis effect of tumor-specific CTL and the concentration of IFN-γ were tested; (**F**) the percentage of CD3^+^CD8^+^T cells from flow cytometry represented the level of specific cellular immune response; (**G**) the percentage of CD3^+^CD8^+^ T cells infiltrated in the tumors was detected by flow cytometry, (* *p* < 0.05; ** *p* < 0.01; *** *p* < 0.001).

The spleen is well acknowledged as an important immune organ. As a proliferation site of lymphocyte, it can partly reflect the immune ability. To determine the immune function of the mice, their spleens were isolated. Measuring the spleen weight revealed that the spleen weight of the combined group was significantly heavier than that of the two single-vaccine groups (*p* < 0.05 for both) (Figure 6A,B). The spleen index in the combined group was the highest compared with that in the DC-CT26 group and the induced HUVEC group (*p* < 0.01 for both) (Figure 6C). Meanwhile, the CTL killing assay and IFN-γ test revealed an obviously greater cellular immune response in the combined group than in the DC-CT26 group and the induced HUVEC group (*p* < 0.001 for both) (Figure 6D,E). As is well known, CD3 molecular only exists in the surface of all T lymphocyte, while CD8^+^ T lymphocyte can directly kill tumor cells.

In this study, firstly, flow cytometry was performed to detect the percentage of the CD3^+^CD8^+^ T cells produced in the spleen. The results showed the percentage of the CD3^+^CD8^+^ T cells in the spleen of combined group was more than that of the two single-vaccine groups (*p* < 0.001 for both) (Figure 6F). Because of the limitation of the FACSCalibur, cells with weak fluorescence intensity cannot be shown in the figures. Thus, the values of the cell populations seem not consistent with dots density. Furtherly, the percentage of CD3^+^CD8^+^ T cells infiltrated in tumor tissues was determined. The results showed that the percentage of CD3^+^CD8^+^ T cells infiltrated in the combined group was obviously higher than that of two single-vaccine groups (Figure 6G). Taken together, these results demonstrate a significantly greater immune function of mice immunized with DC-CT26 and induced HUVECs.

## 4. Discussion

Due to the importance of tumor angiogenesis in the progression of solid tumors, vaccines with xenogeneic or syngeneic endothelial cells targeting tumor angiogenesis have been proven effective [8,10,34]. Immunotherapy of tumors with xenogeneic endothelial cells as a vaccine can break the body’s immune tolerance to its own vascular endothelial cells, induce the immune response against its own tumor vessels, destroy the neovascularization, and inhibit the growth of tumors [7,35].

To our knowledge, this is the first study to evaluate the influence of tumor microenvironment on HUVECs in CRC. Although the HUVEC vaccine has been reported to produce a preventive antitumor microvasculature effect in several tumor models [9,29], the morphology, structure, and function of normal vascular endothelial cells are different from those of tumor vascular endothelial cells [36]. Proliferating tumor endothelium highly expresses antigens, while the antigen expression level of healthy tissues is downregulated or absent on quiescent endothelium. Thus, targeting the tumor vasculature can be feasible for vaccination strategies [37]. Tumor endothelial marker (TEM) is a unique antigenic molecule in tumor vascular endothelial cells (VECs). TEM1 and TEM8 are widely expressed in the vascular system of mouse and human tumor vessels, but the expression in normal adult mouse tissues cannot be detected or can only be detected in a small number of vessels [38,39]. Here, to examine the influence of tumor microenvironment on HUVECs, the supernatant of murine CT26 colorectal cancer cells was applied to simulate the tumor microenvironment. Results showed that the migration and invasion abilities were enhanced in the induced HUVEC group, and the expression levels of TEM1 and TEM8 were also increased in the induced HUVEC group, which revealed that induced HUVECs had characteristics more like tumor vascular endothelial cells than HUVECs (Figure 1). Based on these results, we hypothesized that the antiangiogenesis effect of the induced HUVEC vaccine could be better than that of the HUVEC vaccine.

Results have shown that the induced HUVECs produced a better inhibitory effect on tumor growth and prolonged the survival of tumor-bearing mice (Figure 2). The tumor microvessel CD31 molecular marker is known to be able to accurately reflect the tumor MVD [40,41]. Moreover, the level of angiogenesis in tumor specimens can be examined by the content of hemoglobin [42]. As results showed, the induced HUVEC vaccine produced an obvious inhibition in CD31-positive microvessel density and the content of hemoglobin in tumor tissues (Figure 3A–C). These results indicated that the induced HUVEC vaccine could manifest a better antitumor effect than the HUVEC vaccine by inhibiting tumor angiogenesis. TEM1, TEM8, and VEGFR2 are specific protein molecules that are highly expressed in tumor vascular endothelial cells, promoting tumor angiogenesis [43,44,45]. To investigate the possible mechanism of the antiangiogenic effects of the induced HUVEC vaccine, related antibodies were detected in the serum of mice. The results showed that induced HUVECs effectively produced specific antitumor endothelial cell antibodies in vivo and caused antitumor angiogenesis through humoral immunity (Figure 3D–F). These findings suggested that the induced HUVEC vaccine represented a promising approach in the treatment of CRC.

DC vaccines have recently become a research hotspot in tumor treatment. DC vaccine loading tumor antigen can induce CTLs to kill tumor cells. The tumor cell freeze-thawed antigens contain all the tumor antigens and are widely used [46]. In the present study, murine DCs were loaded with the whole CT26 freeze-thaw antigen. Importantly, there are several advantages of the DC-CT26 vaccine. This vaccine can display all kinds of tumor antigens, stimulate the immune response against these antigens, and avoid the occurrence of immune escape. 

Due to the limitations of the antitumor efficacy of the single DC vaccine, we further investigated a combination approach of the induced HUVEC vaccine with the DC-CT26 vaccine to evaluate antitumor immunity. The results supported that the effect of the combined vaccine on tumor growth was better than that of the single vaccine (Figure 4). Based on CD31-positive microvessel, the combined vaccine exerted a better antitumor angiogenesis effect than the single vaccine (Figure 5A,B). Because IFN-γ can eventually inhibit tumor angiogenesis by inhibiting the proliferation of endothelial cells or indirectly downregulating the release of tumor angiogenic stimuli, mice in the combined group with large amounts of IFN-γ in the serum showed a better antitumor effect (Figure 6E) [47,48,49]. To further analyze the mechanism of antitumor effects in the combined group, western blot and ELISA were performed. The results showed that the levels of specific antiangiogenic-related antibodies in the serum of the combined group were significantly higher than the levels in other groups (Figure 5D,E).

The spleen is well known to be the primary immune organ, exerting an essential role in the immunity of the host. A higher spleen index indicates a stronger immune capability [50,51,52]. Consistent with our findings in antitumor activity, the results showed that the spleen weight in the combined group was higher than that in the other groups (Figure 6A,B). The antitumor mechanism of the combined group was deemed to be the cytotoxic effect of the DC-CT26 vaccine on tumor cells, through inducing CTLs or IFN-γ to kill tumor cells (Figure 6D,E). Interestingly, although the DC-CT26 had the more effective killing effect to CT26 compared with induced HUVEC vaccine in Figure 6D, the difference of their spleen weight was not obvious in 6B. We speculated that as the spleen was a proliferation site of lymphocyte, the spleen weight reflected lymphocyte proliferation capacity. Obviously, the antitumor effect of DC-CT26 was mainly performed by the T lymphocyte, while that of induced HUVEC vaccine was mainly performed by B lymphocyte. Thus, the results of the CTL killing assay, which could reflect the cellular immunity, was different between DC-CT26 vaccine and induced HUVEC vaccine. As the induced HUVEC vaccine might enhance the function of mature DCs, the mature DCs could more effectively activate the initial T cells with the formation of antigenic peptide-MHC class II molecule complexes to produce the killing effect on the target cells [33]. Therefore, the combined vaccine manufactured more CD3^+^CD8^+^ T cells in the spleen (Figure 6F). Previous study has shown that severe CD8^+^ tumor infiltrating lymphocytes (TILs) processed a better prognosis compared with tumors with poor or moderate CD8^+^ TILs infiltration in CRC [53]. In addition, the increase of CD8^+^ TILs within the tumor could contribute to the inhibition of tumor growth [54]. Therefore, we investigated the percentage of CD3^+^CD8^+^ T cells infiltrated in the tumors. Results revealed that the raising percentage of CD3^+^CD8^+^ T cells infiltrated in the combined group led to a longer survival and better antitumor effect compared with the two single-vaccine groups (Figure 6G).

In conclusion, this study demonstrates that the combined vaccine containing induced HUVECs with DC-CT26 elicits strong humoral and cellular immune responses targeting both tumor angiogenesis and tumor cells. The enhanced antitumor efficacy may be attributed to synergistic mechanisms of immune responses against tumor cells as well as tumor microvasculature. As a result, tumor growth was significantly inhibited. This innovative combination of immunotherapeutic approaches may lay the foundation for the clinical treatment of related tumors and provide a new strategy for the development of vaccines against colorectal carcinoma.

## Supplementary Materials

The following are available online at https://www.mdpi.com/2073-4409/8/5/494/s1, Figure S1: 60% human colorectal carcinoma cell supernatant (hormone-depletion) enhanced the migration and invasion abilities of NECs, Figure S2: 60% mouse colorectal carcinoma cell CT26 supernatant (hormone-depletion) enhanced the migration and invasion abilities of NECs.

## Abbreviations

CRC	Colorectal carcinoma
CTLs	Cytotoxic T lymphocytes
DCs	Dendritic cells
DC-CT26	Induced HUVECs and dendritic cell-loading CT26 antigen
FCM	Flow cytometry
H&E	Hematoxylin-eosin
HUVECs	Human umbilical vein endothelial cells
IFN-γ	Interferon-γ
LSD	Least standard difference
MVD	Microvessel density
TCM	Tumor conditioned medium
TEM	Tumor endothelial marker
VECs	Vascular endothelial cells
