# Supplementary material for: Improved Antitumor Efficacy of Combined Vaccine Based on the Induced HUVECs and DC-CT26 Against Colorectal Carcinoma
Source: Cells. 2019 May 22;8(5):494. doi: 10.3390/cells8050494 (PMC6562839; doi:10.3390/cells8050494)
Supplement: Supplementary file 1 [file cells-08-00494-s001.pdf]

Supplementary Materials and Methods

Cell culture

The colorectal cancer cell lines (SW620, HT-29, HCT116, CT26) were obtained from our laboratory. Cells were maintained in DMEM or RPMI-1640 supplemented with 10% FBS (Biological Industries, Kibbutz Beit Haemek, Israel). All cells were maintained at 37 °C with 5% CO_2.

Preparation of tumor conditioned medium (TCM)

The colorectal carcinoma cell lines (SW620, HT-29, HCT116 and CT26) were cultured in 10 cm dishes, and after reaching 60%-80% confluence, Charcoal-stripped FBS (hormone-depletion) (Lot. 1646915, Biological Industries, Kibbutz Beit Heamek, Israel) was applied to exclude the interference of hormone in the serum. After 24 h, the supernatant was collected, centrifuged and stored at -20 °C.

TCM was composed of 60% colorectal carcinoma cell supernatant and 40% ECM medium (with 2% FBS in the medium, without factors and double antibiotics). In the experimental group, NECs (normal endothelial cells) were induced by TCM for 48 h. While in the control group, NECs were induced by FBS free endothelial cell medium at the same condition.

Supplementary Figures

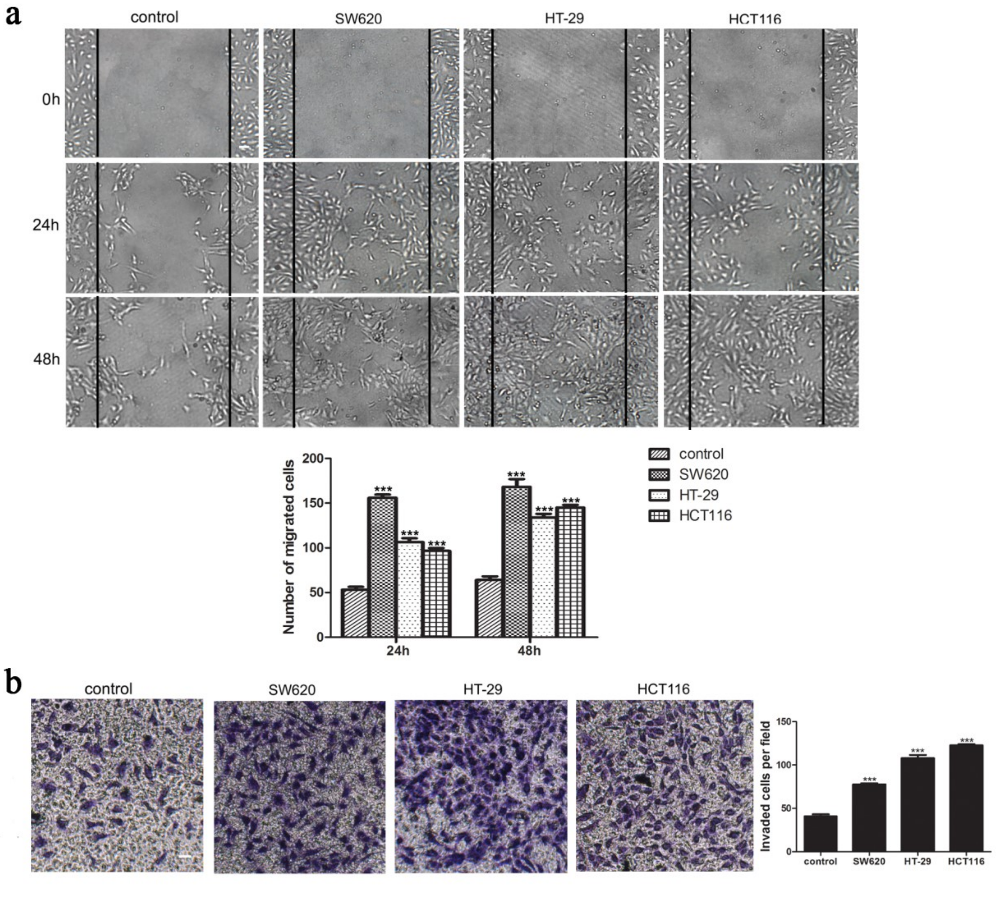

Supplementary Figure S1. 60% human colorectal carcinoma cell supernatant (hormone-depletion) enhanced the migration and invasion abilities of NECs. (A-B) NECs were induced by SW620, HT-29 or HCT116 conditioned medium for 48 h. Then the migration and invasion abilities were examined by wound-healing assay (scale bar 40 μm) (A) and transwell assay (scale bar 20 μm) (B). Data from three independent experiments was presented as mean±SD. (*$p < 0.05$; **$p < 0.01$; ***$p < 0.001$).

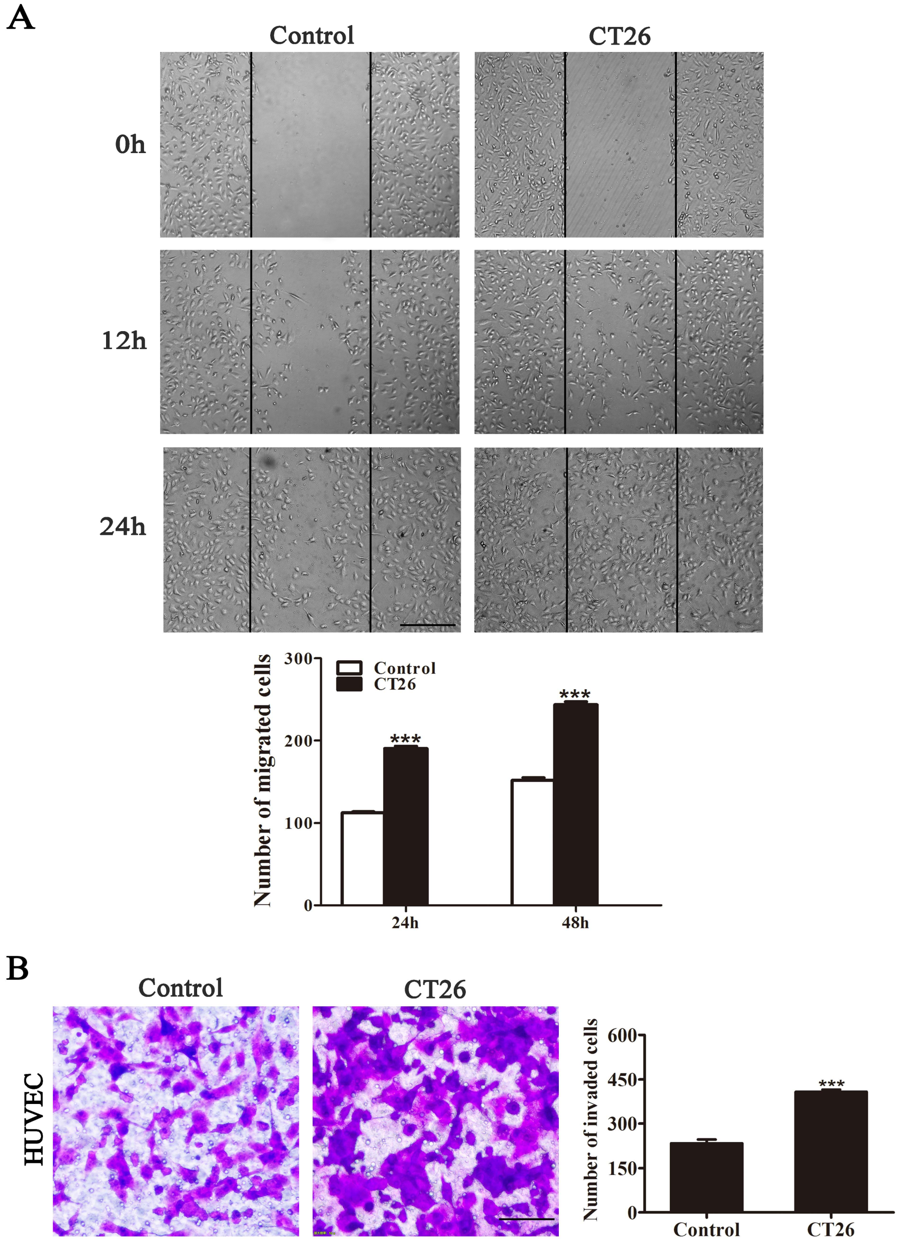

Figure SII

Supplementary Figure S2. 60% mouse colorectal carcinoma cell CT26 supernatant (hormone-depletion) enhanced the migration and invasion abilities of NECs. (A-B) NECs were induced by CT26 conditioned medium for 48 h. Then the migration and invasion abilities were examined by wound-healing assay (scale bar 200 μm) (A) and transwell assay (scale bar 100 μm) (B). Data from three independent experiments was presented as mean±SD. (*$p < 0.05$; **$p < 0.01$; ***$p < 0.001$).
